# A Case of Breast Cancer Following Infliximab Treatment for Treatment-Refractory Crohn’s Disease

**DOI:** 10.5005/jp-journals-10018-1112

**Published:** 2014-07-28

**Authors:** Vedat Goral, Belkis Unsal, Oya Nermin Sivrikoz

**Affiliations:** 1 Department of Gastroenterology, Izmir University Medical Faculty, Izmir, Turkey; 2 Department of Gastroenterology, Izmir Katip Çelebi University Medical Faculty, Izmir, Turkey; 3 Department of Pathology, Izmir Şifa University Medical Faculty, Izmir, Turkey

**Keywords:** Crohn’s disease, Infliximab therapy, Breast cancer.

## Abstract

Crohn’s disease is a chronic, or long lasting inflammatory disease in the gastrointestinal (GI) tract. Most commonly, Crohn’s disease affects the small intestine and the beginning of the large intestine. Treatment for Crohn’s disease usually involves drug therapy or, in certain cases, surgery. Several side effects develop from the use of drugs. A case with Crohn’s disease refractory to 5-ASA, corticosteroid and azathioprine treatments who developed breast carcinoma following infliximab treatment is being presented in this report. Case: SE, aged 44 years, presented to our polyclinic with weight loss, abdominal pain and flatulence. The patient had no response to mesalazine, steroid and azathioprine therapy. Upon identifying inflammatory stricture with abdominal MR, the medicines the patient has been using was discontinued and anti-TNF alpha (infliximab) treatment was initiated after receiving the consent of the patient. At 3rd month of treatment, the patient detected a small mass at the left breast. Mastectomy was performed and axillary lymph nodes were resected.

Because breast cancer was detected following infliximab treatment in this case, we believe that a breast examination (physical examination, mammary USG) must be performed in female patients prior to infliximab therapy.

**How to cite this article:** Goral V, Unsal B, Sivrikoz ON. A Case of Breast Cancer Following Infliximab Treatment for Treatment-Refractory Crohn’s Disease. Euroasian J Hepato-Gastroenterol 2014;4(2): 104-106.

## INTRODUCTION

Crohn’s disease is an inflammatory condition characterized by transmural involvement of the whole gastrointestinal system. Some drugs are used for its treatment, but the treatment is not always simple. Generally, 5-aminosalicylic acid (5-ASA), corticosteroids and immunosuppressive agents are used. Anti-tumor necrosis factor (TNF) antagonists (infliximab, adalimumab) are also prescribed when treatment with former agents proves unsatisfactory. Several side effects develop from the use of infliximab and even malignant conditions including hepatosplenic T-cell lymphoma and skin carcinoma.^1-5^ A case with Crohn’s disease who was refractory to 5-ASA, corticosteroid and azathioprine, and developed breast carcinoma following infliximab treatment is presented in this report.

## CASE REPORT

**Fig. 1:**
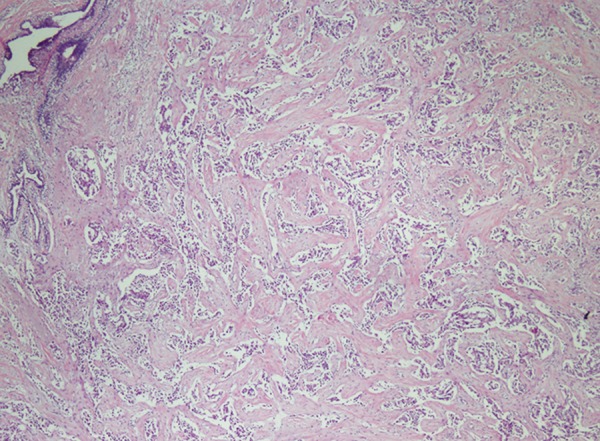
Invasive ductal carcinoma plus ductal carcinoma *in situ* (high grade) (H&E: 40x)

**Fig. 2:**
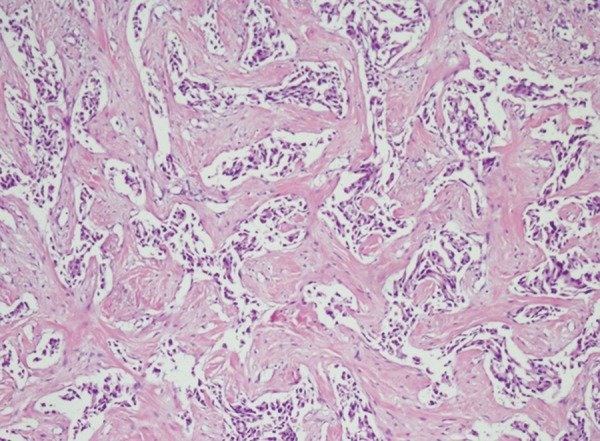
Invasive ductal carcinoma plus ductal carcinoma *in situ* (high grade) (H&E: 100x)

A 44-year-old female patient presented to our polyclinic with weight loss, abdominal pain and flatulence. The patient was under follow-up and treatment for 7 years for Crohn’s disease, with no response to conventional therapy. Upon identifying inflammatory stricture with abdominal magnetic resonance imaging (MRI), the medicines were discontinued and anti-TNF alpha (infliximab) treatment was initiated after receiving the consent of the patient. Quantiferon-TB gold test was performed prior to infliximab treatment. The test was negative and also purified proteing derivative (PPD) test gave a negative response. The patient described that there were no masses or abnormalities in either of the breasts before treatment and anti-TNF was given according to the standard protocol. About 3 months after treatment, the patient detected a small mass at the left breast. Mammography was performed and demonstrated a 15 × 9.5 mm hypoechoic lesion containing millimetric components. Inflamed lymph nodules were detected at the axillary region, the largest of which was 14 mm. A lesion of breast imaging reporting and data system (BIRADS) appearance were identified with double-breast two-way mammography. Invasive ductal carcinoma as well as ductal carcinoma *in situ* (high grade) was detected in the biopsy from this lesion (Figs 1 and 2). The biopsy report shows estrogen receptor (ER) (-) in the invasive tumor; in the *in situ* areas, ER 60% (+), progesterone receptor (PR) (-), C-erbB2 80% (+++), p53 1°% (+) and Ki-67 5%. Mastectomy was therefore performed and axillary lymph nodes were resected. No invasion was identified in the lymph nodes. The patient was then admitted to the general surgery clinic and intestinal resection covering an area of 40 cm including the stricture zone was performed for Crohn’s disease.

## DISCUSSION

Crohn’s disease is a chronic, recurrent condition characterized by transmural inflammation that may affect the whole gastrointestinal tract from the mouth to the anus. Steroids, 5-ASA and immunosuppressive agents (6-mercaptopurine, azathioprine and methotrexate) are used for treatment of Crohn’s disease. However, complete response cannot be achieved always with these agents and because of their side effects, new biological treatments are currently on the agenda. Although the etiology of inflammatory bowel disease (IBD) is not clear, congenital abnormal immune response to luminal bacteria triggers inflammation, which occurs with persisting dysregulation of cellular immunity. Several biological treatments are available (e.g. infliximab, adalimumab, natalizumab and vedolizumab) and some are currently being investigated in clinical trials. Biological agents are effective in cases of corticosteroid-dependent or refractory disease and correct the natural course of the disease. When planning treatment, consideration should be given not only to their potential effects but also to their high cost and potential life-threatening serious complications. Over the past 10 years, anti-TNF agents including inflixi-mab have been emerging as an alternative treatment to overcome the shortcomings of conventional drugs and provide most of IBD patients the improved life quality. Infliximab is an immunoglobulin G1 (IgG1) antibody and is given intravenously. In Crohn’s disease patients, it is recommended as acute induction therapy in three-dose schemes at 5 mg/kg at weeks 0, 2 and 6, followed by the maintenance dose given every 8 weeks. Acute intravenous reaction occurs in 5 to 10% of the cases, but fewer side effects develop with regular administration or when concomitant immunomodulators (azathioprine or methotrexate) are used. The most common reactions are mild to moderate (nausea, headache, dizziness, urticaria, diaphoresis or mild cardiopulmonary symptoms characterized by dyspnea, palpitations, tightness of the chest) and resolve upon slowing down intravenous infusion or with acetaminophen diphenhydramine administration.^3^ Severe reactions (hypotension, severe shortness of breath, tremor and severe chest discomfort) are seen with an incidence of less than 1% and may require oxygen diphen-hydramine, hydrocortisone and epinephrine.^6,7^ Delayed serum sickness-like reactions occur in 1% of the cases.

Breast cancer is a common malignant tumor in women and is a major malignancy resulting in death among women. Genetic alternations, hormonal effects and environmental factors are considered as the major causes leading to its occurrence. A case of breast cancer developing due to infliximab use was presented in this report. There are previous reports, which describe patients developing breast cancer with infliximab use in the literature.^8^ The fact that some patients detect previously unnoticed masses in their breasts after treatment initiation suggests a triggering role of the biological agent used. With this case report, we would like to emphasize the importance of performing a mammary examination and perhaps a mammography before treatment initiation in patients who are to start treatment with biological agents because infliximab has been associated with some malignancies.
